# Electro-Magnetism in a Case of Obstruction of the Bowels

**Published:** 1846-07

**Authors:** 


*Electro-Magnetism in a Case of Obstruction of the Bowels.* The object of the following case is to show the effects, apparently and probably really produced by electro-magnetism. The patient, a widow lady of about 55 years of age, was taken, ten days previous to my seeing her, with bilious vomiting and intense pain in the bowels, accompanied by constipation. The pain ceased in three or four days, but the vomiting continued, and was now stercoraceous. She had been bled to faintness, had taken calomel until the gums were sore, had taken one drop of croton oil every hour, for several doses, besides various other cathartics, and repeated ordinary injection. I found her on the 11th day, without fever, bowels somewhat tender, and vomiting fæcal matter every hour or two. The injections before used had been given with a common syringe and allowed to come away immediately. I used the rectum tube and force pump, but could not throw up more than a quart at once, which, however, was compelled to be retained for half an hour, when it was suffered to pass, and after a little repeated. This was continued for six times, until the injection, which at first returned mixed with fæcal matter, came away, after being kept up an hour, almost as clear as when given. The symptoms nearly the same; there was evident obstruction, and, probably, invagination of the small intestines. I now used Mr. Pike's electro-galvanic apparatus, applying a pole on either side of the abdomen; this was continued for twenty minutes, when there was evident commotion of the bowels, and in ten minutes more was a regular evacuation, thin and small in quantity, but distinctly of a fæcal character and the first in 11 days. The vomiting ceased entirely; two hours after, the battery was again used, and in twenty minutes produced a free operation. A dose of oil operated in the usual time, and the patient rapidly recovered. There was no doubt in my mind but the favorable change was produced by the electro-magnetism, though more probably by the successive shocks producing muscular action, rather than by electro or magnetic agency.—*N. Y. Jour. Med.*

